# Inferring population dynamics of HIV-1 subtype C epidemics in Eastern Africa and Southern Brazil applying different Bayesian phylodynamics approaches

**DOI:** 10.1038/s41598-018-26824-4

**Published:** 2018-06-08

**Authors:** Daiana Mir, Tiago Gräf, Sabrina Esteves de Matos Almeida, Aguinaldo Roberto Pinto, Edson Delatorre, Gonzalo Bello

**Affiliations:** 1 Laboratório de AIDS e Imunologia Molecular, Instituto Oswaldo Cruz, Fiocruz, Rio de Janeiro, Brazil; 2 Departamento de Genética, Instituto de Biologia, Universidade Federal do Rio de Janeiro, Rio de Janeiro, Brazil; 3 KwaZulu-Natal Research Innovation and Sequencing Platform (KRISP), College of Health Sciences, University of KwaZulu-Natal, Durban, South Africa; 4 Centro de Desenvolvimento Científico e Tecnológico, Fundação Estadual de Produção e Pesquisa em Saúde, Porto Alegre, Brazil; 5 Programa de Pós-Graduação em Genética e Biologia Molecular, Universidade Federal do Rio Grande do Sul, Porto Alegre, Brazil; 6 Instituto de Ciências da Saúde, Universidade Feevale, Novo Hamburgo, Brazil; 7 Laboratório de Imunologia Aplicada, Departamento de Microbiologia, Imunologia e Parasitologia, Universidade Federal de Santa Catarina, Florianópolis, Brazil

## Abstract

The subtype C Eastern Africa clade (C_EA_), a particularly successful HIV-1 subtype C lineage, has seeded several sub-epidemics in Eastern African countries and Southern Brazil during the 1960s and 1970s. Here, we characterized the past population dynamics of the major C_EA_ sub-epidemics in Eastern Africa and Brazil by using Bayesian phylodynamic approaches based on coalescent and birth-death models. All phylodynamic models support similar epidemic dynamics and exponential growth rates until roughly the mid-1980s for all the C_EA_ sub-epidemics. Divergent growth patterns, however, were supported afterwards. The Bayesian skygrid coalescent model (BSKG) and the birth-death skyline model (BDSKY) supported longer exponential growth phases than the Bayesian skyline coalescent model (BSKL). The BDSKY model uncovers patterns of a recent decline for the C_EA_ sub-epidemics in Burundi/Rwanda and Tanzania (R_e_ < 1) and a recent growth for Southern Brazil (R_e_ > 1); whereas coalescent models infer an epidemic stabilization. To the contrary, the BSKG model captured a decline of Ethiopian C_EA_ sub-epidemic between the mid-1990s and mid-2000s that was not uncovered by the BDSKY model. These results underscore that the joint use of different phylodynamic approaches may yield complementary insights into the past HIV population dynamics.

## Introduction

The human immunodeficiency virus type 1 (HIV-1) subtype C accounts for approximately 48% of all people living with HIV, representing the most prevalent HIV-1 subtype in the world^1^. The high global prevalence of the C subtype results from its predominance in regions with the highest rates of HIV-1 infection and with large populations, such as Southern and Eastern Africa, India and Southern Brazil^1,2^. The origin of HIV-1 subtype C was recently traced to the Katanga region of the Democratic Republic of Congo (DRC) in the late 1930s^3^ from where it spread independently to Eastern and Southern Africa, leading to a phylogeographic subdivision between the HIV-1 subtype C strains circulating in those two African regions^4,5^.

The expansion of HIV-1 subtype C inside Eastern Africa gave rise to the C East African clade (C_EA_), whose most probable epicenter of dissemination was in Burundi around the early 1960s. During the 1970s, this country acted as ignition point of several local C_EA_ sub-epidemics in other Eastern African countries^5^ and also in Southern Brazil^6^ where the C_EA_ sub-epidemic was fueled from a single founder event^7^. The C_EA_ clade currently predominates among subtype C strains from Eastern African countries and Brazil, and accounts for almost 100% of subtype C sequences from Burundi and Brazil, 97% from Uganda, 64% from Kenya, 61% from Ethiopia and 49% from Tanzania^5,8^. The evolutionary analyses of the C_EA_ sub-epidemics performed so far mostly address questions about the place and timing of outbreaks onset, focusing on the reconstruction of the geographic dissemination pathways of this viral clade^2,5,6,8–12^. Studies on the population dynamics of C_EA_ sub-epidemics, while key to understand their historical epidemic growth trends, epidemic potential and ecological processes shaping their evolution, have been much less frequent^13,14^.

Key epidemiological and population parameters, most notably the effective number of infections (*Ne*), the epidemic growth rate (*r*) and the basic (R_0_) and effective (R_e_) reproductive number, can be estimated from viral sequence data by using Bayesian phylodynamic approaches based on coalescent^15^ and birth-death^16^ models. These models have very different mathematical grounds as well as particular strengths and limitations. The coalescent is appropriate only if the number of sampled infected individuals is small compared with the size of the total infected population^16^, despite certain robustness to violation of this requirement has been demonstrated^17^. The birth-death model, meanwhile, explicitly models the sampling process and can thus be used for sparse or densely sampled viral populations^16^, although estimates may be biased if the model of the sampling process is misspecified. The coalescent allows the inference of the R_0_ (key epidemiological parameter indicator of increment [R_0_ > 1], decline [R_0_ < 1] or stabilization [R_0_ = 1] in the number of new cases^18^) only through modeling the population dynamics under a deterministic assumption, which represents a limitation for populations undergoing complex dynamics^19^, and require an independent estimate of the average duration of infectiousness. The birth–death model has the advantage of accounting for stochasticity of the demographic process and provides an estimate for R_e_ changes over time using only sequence data^16,20^. A potential disadvantage of the birth-death model is that credibility intervals grow wider the further we go into the past, which is not the case for the coalescent-based models^21^; although simulation studies showed that the coalescent might not capture the true *r* because of the narrow credibility intervals around the median estimate attributed to its assumption of deterministic changes in the population size^20^.

The present work aims to shed light on the past population dynamics of the major HIV-1 C_EA_ sub-epidemics established in Burundi, Rwanda, Ethiopia, Tanzania and Brazil by analyzing viral *pol* gene sequences sampled between 1990 and 2014 with Bayesian phylodynamic methods based on coalescent and birth-death models.

## Materials and Methods

### Sequence dataset compilation

A reference dataset of HIV-1 subtype C *pol* sequences belonging to the east, southern and central African lineages was selected from a previous study^5^ and combined with: 1) more recent east African subtype C *pol* sequences with known sampling dates available in Los Alamos HIV Database (http://www.hiv.lanl.gov) by August 2017, and 2) subtype C *pol* sequences with known sampling dates isolated from heterosexual populations living in the two southernmost Brazilian states (Rio Grande do Sul and Santa Catarina) previously described^11^. The option “One sequence/patient” was selected from Los Alamos HIV database to exclude multiple sequences from the same subject. The subtype assignment of all sequences was confirmed using the REGA HIV-1 subtyping tool v.3.0. Given the two genetically distinct subtype C clades (C and C’) co-circulating in Ethiopia^22^, linked to subtype C viruses of eastern and southern African origin respectively, putative intrasubtype C/C’ recombinant sequences (*n* = 99) were identified by Bootscanning using Simplot v3.5.1^23^ as described previously^5^ and removed from further analyses. This resulted in a final dataset of 1,147 HIV-1 subtype C *pol* sequences (Table S1) covering the complete protease (PR) and the first part of the reverse transcriptase (RT) regions (nucleotides 2,253 to 3,272 relative to HXB2 genome).

### Identification of dominant country-specific HIV-1 C_EA_ subclades

To identify major country-specific clades within the C_EA_ radiation, HIV-1 subtype C *pol* sequences from eastern Africa and southern Brazil were first aligned with reference subtype C sequences belonging to the eastern, southern and central African clades using the CLUSTAL X program^24^ and subjected to maximum likelihood (ML) phylogenetic analysis. ML trees were inferred with the PhyML program^25^, using an online web server^26^, under the general time-reversible model of nucleotide substitution plus invariant sites and four discrete gamma rate categories (GTR+I+Γ4) selected with jModeltest program^27^ and the subtree pruning and regrafting (SPR) branch-swapping algorithm of heuristic tree search. The reliability of the phylogenies was estimated with the approximate likelihood-ratio test based on a Shimodaira–Hasegawa-like procedure (SH-*aLRT*)^25^. Basal HIV-1 C_EA_ sequences from Burundi and Rwanda and major (n ≥ 50 sequences) country-specific (>90% of sequences from a single country) monophyletic groups with high support (SH-*aLRT* ≥0.85) nested within the C_EA_ clade radiation were selected for demographic analyses. Reference sequences of HIV-1 subtypes A1 and D from the Los Alamos HIV Database were used as outgroups. Final trees were visualized in FigTree v1.4.2.

### Estimation of phylodynamic parameters

Epidemiological and evolutionary parameters of the defined C_EA_ subclades were estimated via Bayesian Markov Chain Monte Carlo (MCMC) phylogenetic inference using coalescent and birth-death tree priors as implemented in BEAST v1.8^28^ and BEAST v.2.4^29^ software packages, respectively. Changes in *Ne* using the coalescent tree prior were first assessed using the non-parametric Bayesian skyline (BSKL)^30^ and Bayesian Skygrid (BSKG)^31^ models and estimates of the *r* were subsequently obtained using the parametric model that provided the best fit to the demographic signal contained in each dataset. Comparison between demographic models (logistic, exponential, or expansion) was performed using the log marginal likelihood estimation (MLE) based on path sampling (PS) and stepping-stone sampling (SS) methods^32^. The cumulative number of lineages through time (LTT) was calculated from the combined posterior distribution of sampled coalescent tree topologies by using TRACER v1.6 program^33^. A special case of the birth-death tree prior, namely the birth-death skyline (BDSKY) was applied to model viral transmissions through time^21^. The sampling rate (δ) was set to zero for the period prior to the oldest sample and estimated from the data afterwards. The R_e_ was estimated in a piece-wise manner over three different equidistant intervals using a lognormal prior distribution (R_e_: mean = 0, standard deviation = 1). Bayesian analyses for each transmission clade employed the GTR+I+Γ4 model of nucleotide substitution selected using the jModelTest program^27^ and a relaxed uncorrelated lognormal molecular clock model^34^. Because linear regression analysis of root-to-tip distances as function of sampling time obtained by TempEst v1.5^35^ revealed low temporal signal in the datasets, an informative normal prior distribution on the time to the most recent common ancestor (tMRCA) was applied based on previous estimates^5,6^. MCMC chains were run for sufficiently long to ensure stationarity (constant mean and variance of trace plots) and good mixing (Effective Sample Size >200) for all parameter estimates, as diagnosed by TRACER v1.6 program^33^.

## Results

### Identification of major subclades within the HIV-1 C_EA_ clade radiation

To obtain a more updated picture of the HIV-1 C_EA_ clade radiation, subtype C *pol* sequences from Eastern Africa and Southern Brazil deposited in Los Alamos HIV sequence database between 2013 and 2016 were combined with C_EA_ *pol* sequences from those regions previously characterized^5,6^. The reconstructed ML phylogeny showed that most (79%) subtype C sequences from Eastern Africa and all sequences from Southern Brazil sampled at most recent time (2013–2016) branched within the highly supported (SH-*aLRT* = 0.96) HIV-1 C_EA_ clade (Fig. S1). As expected, sequences from Burundi and Rwanda were highly intermixed among each other and occupied the most basal positions of the C_EA_ clade radiation; while sequences from other Eastern African countries and Brazil were nested within Burundian and Rwandan C_EA_ sequences.

**Figure 1:**
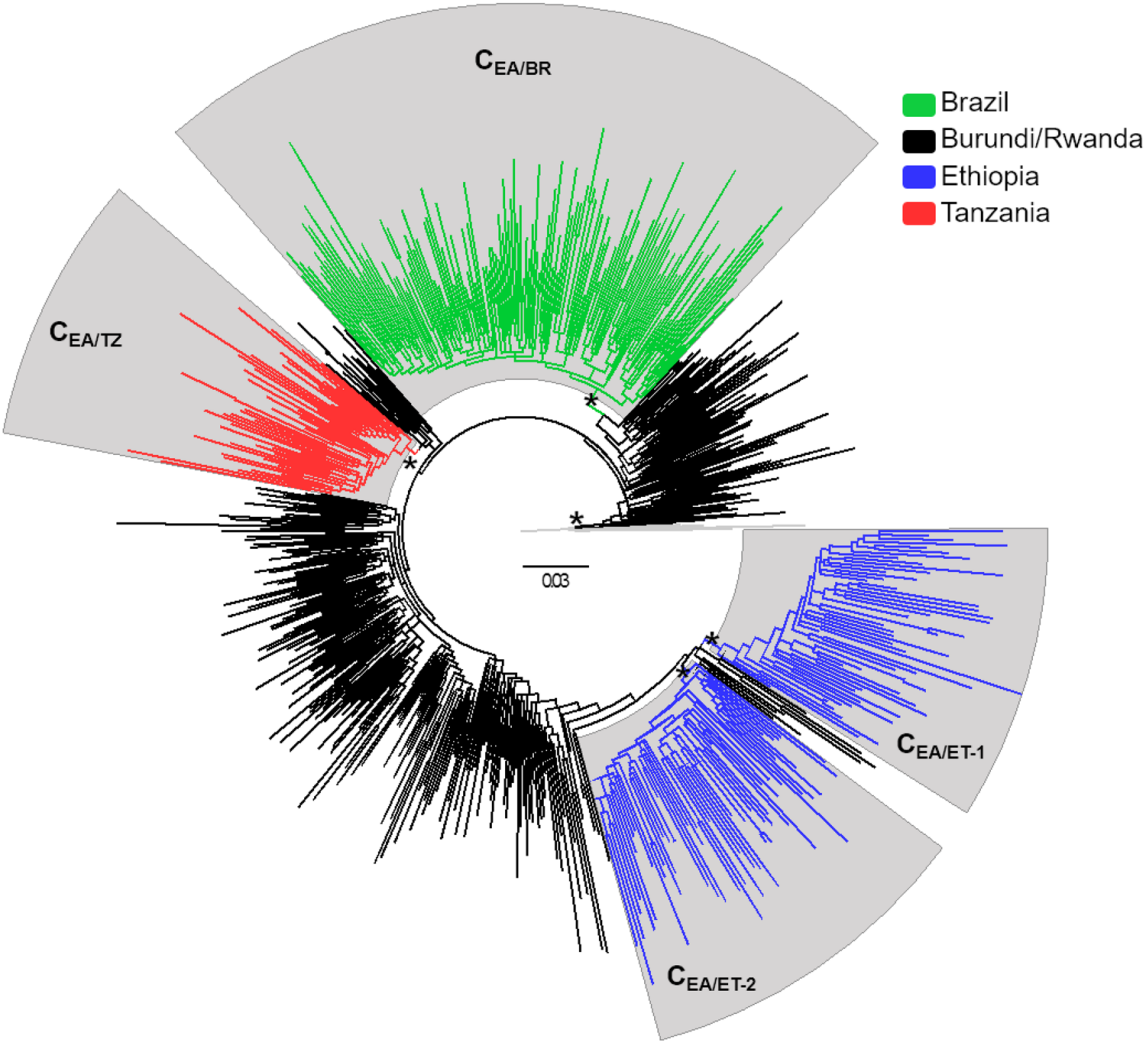
ML phylogenetic tree of HIV-1 C*EA pol* PR/RT sequences (~1,000 nt) from eastern Africa and southern Brazil. Branches are colored according to the geographic origin of sequences as indicated in the legend (upper right). Gray shaded boxes indicate the positions of major C_EA_ lineages. Asterisks point to key nodes with high support (SH-*aLRT* >0.85). The tree was rooted using HIV-1 subtypes A1 and D reference sequences and the branch lengths are drawn to scale with the bar at the center indicating nucleotide substitutions per site.

Most sequences from Kenya and Uganda appeared as sporadic (non-clustered) lineages or clustered in monophyletic subclades of small sizes (*n* < 50) (Fig. S1). All Brazilian sequences and most sequences from Ethiopia (67%) and Tanzania (66%), by contrast, branched within four country-specific C_EA_ subclades of large size (*n* > 50) (Fig. S1) that were more clearly visualized after pruning of non-clustered C_EA_ sequences and C_EA_ sequences within monophyletic subclades of small sizes (Fig. 1). The four identified C_EA_ subclades (C_EA/BR_, C_EA/ET-1_, C_EA/ET-2_ and C_EA/TZ_) together with sequences from Burundi and Rwanda (C_EA/BI-RW_) comprise 76% (*n* = 616) of all the C_EA_ sequences analyzed here; thus confirming the epidemiological relevance of the selected subclades.

### Bayesian population dynamics inference in a coalescent framework

**Figure 2:**
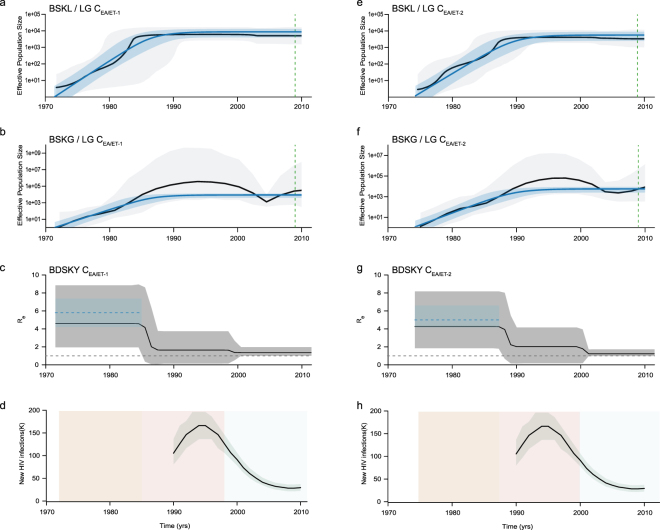
Epidemiological and population dynamics of the C_EA_ sub-epidemics in Ethiopia. Median estimates of the effective number of infections (*Ne*) using the Bayesian skyline or skygrid models (black lines) together with their 95% highest probability density (HPD) intervals (gray areas), co-plotted together with the median *Ne* estimates using the logistic coalescent-based parametric model (blue lines) and its 95% HPD intervals (blue areas). The green dashed lines indicate the time of the last coalescent event reported by the lineages-through-time (LTT) (**a**,**b**,**e**,**f**). Temporal fluctuation of the effective reproductive number (R_e_) of the C_EA/ET-1_ and C_EA/ET-2_ sub-epidemics estimated using the Bayesian birth-death approach (**c** and **g**). For an easier visualization, the median coalescent-based R_0_ estimate (blue dashed lines) inferred for each sub-epidemic and its 95% HPD intervals (blue area) were added. The gray dashed lines indicate R_e_ = 1 (**c** and **g**). Plots representing the number of new HIV cases in Ethiopia as obtained from UNAIDS website http://aidsinfo.unaids.org/ (**d** and **h**). The yellow, pink and gray intervals denote the time spanned for the birth-death-based R_e_-initial, R_e_-middle and R_e_-final estimates of each C_EA_ sub-epidemic.

**Figure 3:**
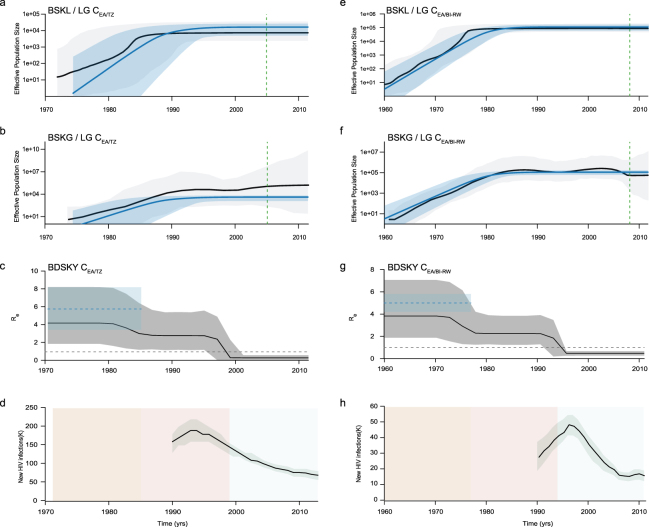
Epidemiological and population dynamics of the C_EA_ sub-epidemics in Tanzania and Burundi/Rwanda. Median estimates of the effective number of infections (*Ne*) using the Bayesian skyline or skygrid models (black lines) together with their 95% highest probability density (HPD) intervals (gray areas), co-plotted together with the median *Ne* estimates using the logistic coalescent-based parametric model (blue lines) and its 95% HPD intervals (blue areas). The green dashed lines indicate the time of the last coalescent event reported by the lineages-through-time (LTT) (**a**,**b**,**e**,**f**). Temporal fluctuation of the effective reproductive number (R_e_) of the C_EA/TZ_ and C_EA/BI-RW_ sub-epidemics estimated using the Bayesian birth-death approach (**c** and **g**). For an easier visualization, the median coalescent-based R_0_ estimate (blue dashed lines) inferred for each sub-epidemic and its 95% HPD intervals (blue area) were added. The gray dashed lines indicate R_e_ = 1 (**c** and **g**). Plots representing the number of new HIV cases in Tanzania and Burundi/Rwanda as obtained from UNAIDS website http://aidsinfo.unaids.org/ (**d** and **h**). The yellow, pink and gray intervals denote the time spanned for the birth-death-based R_e_-initial, R_e_-middle and R_e_-final estimates of each C_EA_ sub-epidemic.

**Figure 4:**
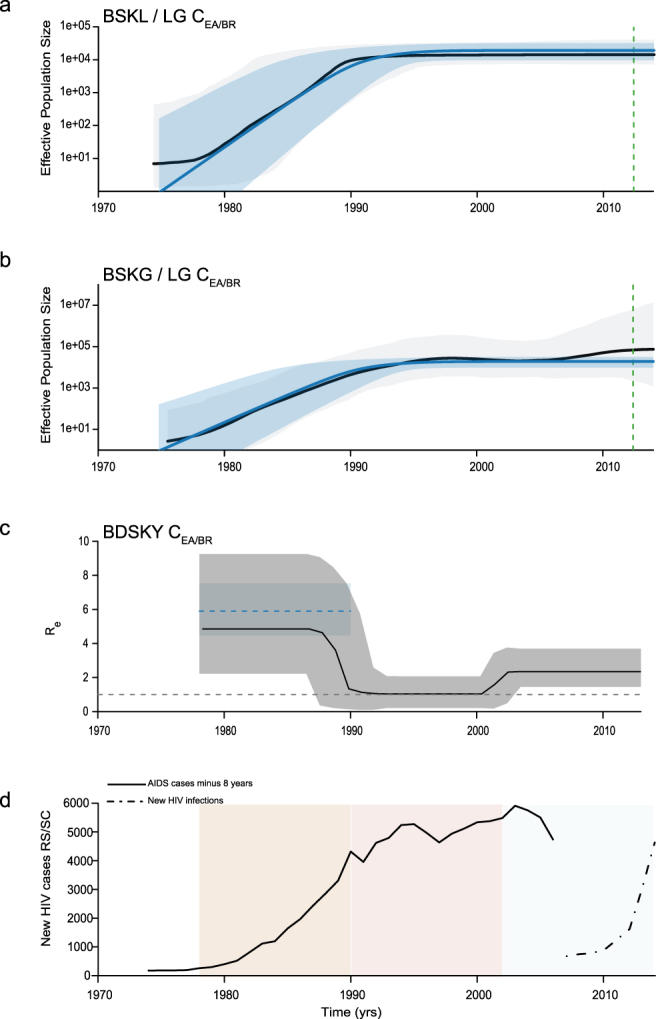
Epidemiological and population dynamics of the C_EA_ sub-epidemic in southern Brazil. Median estimates of the effective number of infections (*Ne*) using Bayesian skyline or skygrid models (black lines) together with their 95% highest probability density (HPD) intervals (gray areas), co-plotted together with the median *Ne* estimates using the logistic coalescent-based parametric model (blue lines) and its 95% HPD intervals (blue areas). The green dashed lines indicate the time of the last coalescent event reported by the lineages-through-time (LTT) (**a** and **b**). Temporal fluctuation of the effective reproductive number (R_e_) of the C_EA/BR_ sub-epidemic estimated using the Bayesian birth-death approach (**c**). For an easier visualization, the median coalescent-based R_0_ estimate (blue dashed line) inferred for the C_EA/BR_ subclade and its 95% HPD intervals (blue area) were added. The gray dashed line indicate R_e_ = 1 (**c**). Plot representing the number of new HIV cases in the Southern Brazilian states of Rio Grande do Sul (RS) and Santa Catarina (SC). AIDS cases reported by the Brazilian AIDS cases databank (SINAN = SIM = SISCEL: http://www.portalsinan.saude.gov.br/dados-epidemiologicos-sinan), minus eight years, was used as an approximation for new HIV infections (solid black line). From 2007 onward, Brazilian Ministry of Health started to report HIV new infections (http://www.aids.gov.br/pt-br/pub/2016/boletim-epidemiologico-de-aids-2016), reprented here as a dashed black line (**d**). The yellow, pink and gray intervals denote the time spanned for the birth-death-based R_e_-initial, R_e_-middle and R_e_-final estimates of the C_EA/BR_ sub-epidemic.

Bayesian MCMC coalescent-based analyses under the BSKL model suggest that all C_EA_ subclades presents roughly comparable demographic histories, with an initial phase of fast exponential growth followed by a stabilization of the *Ne* at some time between the late 1970s and the late 1980s that persisted until the most recent sampling date of each of them (Figs 2a,e, 3a,e and 4a). The observed stabilization in the *Ne* of African C_EA_ sub-clades occurs before the respective stabilization of the HIV incidence in corresponding countries estimated around the mid-1990s according to the UNAIDS (Figs 2d,h and 3d,h). The UNAIDS data also supports a significant reduction of the HIV incidence in Burundi/Rwanda, Ethiopia and Tanzania between the mid-1990s and the mid-2000s that was not captured by the BSKL inference. The stabilization of the C_EA/BR_ *Ne* around the early 1990s is consistent with the stabilization of the new HIV cases in Rio Grande do Sul and Santa Catarina at around the same time (considering a lag time of eight years between HIV infection and new AIDS cases reported by the Brazilian AIDS cases databank for those Brazilian states); but fails to capture a recent increase in the number of new HIV cases from 2010 onwards (Fig. 4d).

The BSKG demographic reconstructions point to a longer period of exponential growth for all C_EA_ subclades that extends up to between the early and the mid-1990s, in agreement with incidence data. For the Ethiopian C_EA_ sub-epidemics, the BSKG points to subsequent decline of the median *Ne* until mid-2000s and a final plateau until 2010, in agreement with epidemiological data (Fig. 2b,d,f,h). The median estimated *Ne* for the C_EA/BI-RW,_ C_EA/TZ_ and C_EA/BR_ sub-epidemics reach a plateau that persisted until the most recent sampling time which differs markedly from the HIV incidence pattern (Figs 3b,d,f,h and 4b,d). The large 95% highest probability density (HPD) interval of the *Ne* estimates inferred by the BSKG model, however, may accommodate different demographic patterns in the latter stages making it difficult to draw solid conclusions on the consistency (or the lack thereof) between these estimates and the HIV incidence temporal pattern.

The logistic growth model was the best-fit parametric model of population growth for all the C_EA_ subclades (Table S2). The median *Ne* trajectories obtained by the logistic growth model during the initial exponential phase closely matched the corresponding trajectories obtained with the non-parametric approaches, particularly those obtained with the BSKG (blue line Figs 2a,b,e,f, 3a,b,e,f and 4a,f). The mean R_0_ values derived from the logistic growth model using the formula R_0_ = *r*D + 1^15^ (where D is average duration of infectiousness herein considered of eight years) was very similar for all C_EA_ subclades, ranging between 5.0 and 5.9 (Table 1). The median *Ne* trajectories of the C_EA/BI-RW_, C_EA/TZ_ and C_EA-BR_ at the plateau phase obtained with parametric and non-parametric approaches were very similar. As the logistic growth model is unable to capture non-constant trends on recent time period estimates, obviously do not reproduced the declining phase pointed by the BSKG for the C_EA/ET-1_ and C_EA/ET-2_ sub-clades, however, the parametric and non-parametric models converge to similar median *Ne* values at the late plateau phase.

**Table 1:** Evolutionary and demographic parameters estimated for HIV-1 C_EA_ subclades.

Clade	N	Sampling interval	Method	Substitution rate (10^−3^)	T_MRCA_	R_0_/Re-initial	Re-middle	Re-final
BI-RW	303	2002–2012	coalescent (logistic)	1.5 (1.3–1.6)	1958 (1952–1964)	5.0 (4.2–5.8)	—	—
			birth-death	1.5 (1.4–1.6)	1957 (1952–1962)	3.8 (1.9 –7.1)	2.3 (1.2–3.8)	0.5 (0.3–0.7)
ET-1	63	2003–2011	coalescent (logistic)	1.2 (1.1–1.4)	1971 (1969–1973)	5.8 (4.2–7.4)	—	—
			birth-death	1.3 (1.0–1.5)	1972 (1970–1974)	4.6 (2.0–8.8)	1.6 (0.1–3.7)	1.3 (1.0–2.0)
ET-2	56	2003–2012	coalescent (logistic)	1.1 (0.9–1.3)	1974 (1972–1976)	5.0 (4.2–6.6)	—	—
			birth-death	1.1 (1.0–1.3)	1974 (1973–1976)	4.3 (1.9–8.1)	2.0 (0.2–4.2)	1.2 (1.0–1.7)
TZ	50	2004–2014	coalescent (logistic)	1.2 (0.8–1.7)	1974 (1963–1984)	5.8 (3.4–8.2)	—	—
			birth-death	1.1 (0.9–1.3)	1971 (1966–1977)	4.2 (1.8–8.2)	2.8 (1.2–5.4)	0.3 (0.1 –0.6)
BR	144	1992–2014	coalescent (logistic)	1.6 (1.2–2.0)	1974 (1966–1982)	5.9 (4.4–7.6)	—	—
			birth-death	1.5 (1.3–1.7)	1978 (1974–1981)	4.9 (2.2–9.2)	1.0 (0.2–2.1)	2.4 (1.4–3.7)

*The 95% credibility intervals for all estimates are indicated inside parenthesis.

### Bayesian population dynamics inference under birth-death model

The BDSKY model was applied allowing the R_e_ to change in a piece-wise manner over three different time intervals that allow us to estimate the epidemic potential before identification of HIV (first time interval, ~1960–1970 to 1980–1984) and observe the potential impact of prevention (second time interval, ~1981–1985 to 1995–1999) or therapy (third time interval, ~1996–2000 to 2012–2014) measures on epidemic dynamics (Figs 2c,g, 3c,g and 4c). The BDSKY model support initial exponential growth dynamics fully consistent with those estimated using coalescent models. Although the mean R_0_ values (5.0–5.9) were slightly higher than the estimated mean R_e_-initial (3.8–4.9), the uncertainty on the R_0_ estimates was always contained within the broader 95% HPD intervals of R_e_-initial (Table 1). The BDSKY model supports a progressive reduction of the mean R_e_ in the second (mean R_e_-initial >mean R_e_-middle) and third (mean R_e_-middle >mean R_e_-final) time intervals for all African subclades. Although the mean R_e_ estimates for the Ethiopian clades remained above one during all time intervals, which clearly did not match with the declining and subsequent stabilization of the HIV incidence in Ethiopia from the mid-1990s onwards (Fig. 2d,h), those R_e_ estimates should be interpreted with caution because the extremely wide 95% HPD intervals (Table 1). For the C_EA/BI-RW_ and C_EA/TZ_ subclades, the R_e_-middle was above one, while the R_e_-final was below one, in agreement with the increasing HIV incidence in Burundi/Rwanda and Tanzania up to the mid-1990s and the subsequent declining from the mid-1990s onwards (Fig. 3d,h). For the C_EA/BR_ subclade, the BDSKY model supports a reduction of the mean R_e_ in the second interval (R_e_-initial ~1) and a new increase in the third one (R_e_-final >1). This is consistent with the HIV incidence trends in Rio Grande do Sul and Santa Catarina Brazilian states, that supports an epidemic stabilization during the 1990s and a new epidemic increase during the 2000s (Fig. 4c,d).

## Discussion

In this study, we characterized key features of the epidemic dynamics of major HIV-1 subtype C_EA_ lineages circulating in East Africa and Southern Brazil through the use of different phylodynamic frameworks based in coalescent and birth-death process. The different coalescent models capture very similar epidemic dynamics over the earlier decades of the C_EA_ lineages dissemination; but point to quite different epidemic dynamics from the mid-1980s onwards.

Both phylodynamic approaches suggest an initial stage of fast exponential growth of all the C_EA_ sub-epidemics during the period of cryptic transmission of HIV in human populations. These initial phases of exponential growth herein inferred correlate with retrospective serological-based studies and simulations that indicated that an explosive epidemic was already sweeping the Eastern African region^36–41^ by 1981, when the AIDS was first recognized. The exponential growth phase inferred for the Brazilian C_EA_ sub-epidemic during the 1970s and 1980s is also fully consistent with the sharp increase in the number of new HIV cases detected in the southernmost Brazilian states during the 1980s^42^. The mean R_0_ (5.0–5.9) and R_e_-initial (3.8–4.9) values here estimated for all C_EA_ sub-clades were roughly comparable. The 95% HPD intervals of R_0_ were always smaller and contained within the broader 95% HPD intervals of R_e_-initial, consistent with previous empirical and simulated data^20,21^. The coalescent-based logistic growth model is expected to provide narrow HPD intervals because it considers a deterministic population trajectory, while the BDSKY model incorporates stochasticity in population size. Additionally, the HPD interval of R_e_ estimates from the BDSKY model grows wider the further we go towards the past, that is not the case for the coalescent-based logistic growth model estimates.

Importantly, the mean R_0_ and R_e_-initial values inferred for the African C_EA_ sub-epidemics were fully consistent with those estimated through analyses of HIV prevalence rate and life expectancy in Eastern African countries^43^. This suggests that both phylodynamic frameworks were able to recover the true early growth rates of HIV-1 C_EA_ sub-epidemics.

Factors like gender inequality^44–46^, civil and ethnic conflicts^47–50^, conflict-induced displacement, and increasing urbanization^51,52^ have shaped the early HIV epidemic dynamics across all Eastern African countries, consistent with the similar epidemic growth rates of African C_EA_ sub-epidemics. Notably, the epidemic growth rate inferred for the Brazilian C_EA_ sub-epidemic was very similar to those obtained for the African C_EA_ sub-epidemics despite significantly distinctive history of human conflicts in those regions. The exponential growth phase of the C_EA/BR_ sub-epidemic matches with a period in which public health system was unaware about the severity of the epidemic^53^ and the C_EA/BR_ subclade was efficiently disseminated in Southern Brazil through heterosexual (HET) networks^2,13,54^, similar to that observed in Eastern Africa. This suggests that the absence of prevention efforts and the predominant viral transmission through HET route may have been the common driving forces of the early dynamics of the C_EA_ sub-epidemics in Eastern African and Southern Brazil.

The BSKL model supports that African C_EA_ sub-epidemics grew exponentially until between the late 1970s and late 1980s, after which there occurs a plateau in the *Ne* until the most recent sampling time of each of them. The stabilization of the *Ne* trajectories occurs around 10 years before the last coalescent event (Figs 2a,b,e,f and 3a,b,e,f), thus supporting that the inferred plateau of the *Ne* is not due to a paucity of coalescent events after the early 1990s^55^. More important, such stabilization occurred before implementation of prevention campaigns during the 1990s^56–60^ and introduction of universal access to antiretroviral (ARV) therapy during the 2000s^61,62^ in Eastern Africa. The overall *Ne* trajectories inferred by the BSKL after the mid-1980s, however, differ markedly from the data of the United Nations Joint Program on HIV/AIDS (UNAIDS)^63^ according to which the HIV incidence in Burundi/Rwanda, Ethiopia and Tanzania reached a peak around the mid-1990s (rather than during the 1980s), and was followed by a sharp decline (rather than a plateau) until the mid-2000s, before stabilize.

The overall epidemic dynamics inferred by the BSKG model from the mid-1980s to the mid-1990s are more consistent with the HIV incidence data than those inferred by the BSKL, although some divergences were also detected at later times. The BSKG model points that C_EA_ African sub-epidemics grew exponentially until the early/mid-1990s and further supports a declining *Ne* of C_EA/ET_ subclades between the mid-1990s and the mid-2000s, consistent with epidemiological data. This model, however, failed to capture a similar decline of *Ne* for the C_EA/BI-RW_ and C_EA/TZ_ sub-epidemics. These results indicate that the BSKG model can correctly predict epidemic decline in some situations, as demonstrated here for the C_EA/ET_ subclades and previously for the CRF02_AG epidemic in Cameroon^31,64^; but not in others.

Although the BDSKY model also supports a progressive reduction of epidemic growth over time, an R_e_ >1 was estimated at the second time interval (that roughly covers the period between early/mid-1980s and mid/late 1990s) for all African C_EA_ subclades, consistent with a continuous increase of HIV incidence up to the mid-1990s. The BDSKY model capture an R_e_ <1 for the C_EA/BI-RW_ and C_EA/TZ_ sub-epidemics at the most recent time interval (after the mid-1990s); but supported an R_e_ ≥1 for the C_EA/ET_ sub-epidemics in the same time interval. These results confirms that the BDSKY model can correctly uncover a signature of a recent declining epidemic not reflected in the coalescent plots, as previously seen in the HIV-1 subtype B epidemic in the UK^21^; but also reveals that it may fail to capture such trend in some other datasets.

It is interesting to note that the BSKG failed to capture a decline of *Ne* for the C_EA/BI-RW_ and C_EA/TZ_ sub-epidemics since the middle-late 1990s onwards, while the BDSKY model failed to capture a R_e_ ≤1 for the C_EA/ET_ sub-epidemics at the same time interval, suggesting that the performance of different phylodynamics approaches could be affected by different factors. The BSKG model requires strongly informative data to prevent erroneous estimates of *Ne* stabilization as pointed by a recent study^65^. The lower proportion of HIV-1 subtype C sequences sampled at recent times (since 2008 onwards) in the C_EA/BI-RW_ (6%) and C_EA/TZ_ (30%) datasets compared with the C_EA/ET_ datasets (≥70%) may have reduce the ability of this coalescent model to capture changes in *Ne* for the C_EA/BI-RW_ and C_EA/TZ_ sub-epidemics at most recent times. The BDSKY model could be more robust to the paucity of coalescent events at most recent time; but its performance could be limited by the number of time intervals (changes in R_e_) specified. Increasing the number of R_e_-changes may allow a better fit of the R_e_ trajectories to the epidemiological data for the C_EA/ET_ sub-epidemics. This strategy, however, resulted in a lack of parameter convergence and huge 95% HPD intervals, indicating that accurate R_e_ estimations at more time intervals would require a larger number of C_EA/ET_ sequences than those used in the present study.

The BSKL, BSKG and BDSKY models support quite consistent epidemic dynamics for the C_EA/BR_ sub-epidemic until the late 1990s. According to the coalescent models, the *Ne* of the C_EA/BR_ subclade growth exponentially until the early (BSKL) or mid-1990s (BSKG) and then reached a plateau. In agreement, the BDSKY model supports an expanding epidemic (R_e_ >1) in the first time interval (~ mid-1970s to late 1980s) and a transient epidemic stabilization (R_e_ ~ 1) in the second time interval (~ late 1980s to early 2000s). The stabilization of the C_EA/BR_ incidence since the early/mid-1990s is in line with the reported trend toward stability of the new HIV cases in Rio Grande do Sul and Santa Catarina states since the mid-1990s^42^, probably due to the implementation of prevention efforts that acted as the driven-force of people’s behavioral changes^53,66^. While coalescent models support a roughly constant *Ne* for the C_EA/BR_ sub-epidemic until the most recent sampling time, the BDSKY model uncovers a new epidemic increase (R_e_ >1) at the last time interval. This matches with an upward trend of new HIV diagnoses in Rio Grande do Sul and Santa Catarina states since 2007^67^. Such epidemiological changes are probably too recent to be fully captured by coalescent models.

A recent study using BSKG to analyze the population dynamics of the C_EA/BR_ sub-epidemic from *pol* and *env* sequences from HET and men having sex with men (MSM) individuals reported a continuous increase in the *Ne* until mid to late 2000’s that was associated with the recent expansion of subtype C throughout the MSM group^13^. Interestingly, universal access to free fully suppressive ARV therapy is available in Brazil since the late 1990s^61,62^ and an association between ARV treatment availability and increases in sexual risk behavior (and consequent rise in HIV incidence) have been previously reported among MSM from developed countries^68–71^. Our BDSKY analyses of sequences from HET individuals support that the recent expansion of the C_EA/BR_ sub-epidemic is probably not restricted to a specific group, but also occurred among HET individuals. Increases in sexual risk behavior among HET individuals fully agrees with the sustained increase of HIV^67^ and other sexually transmitted disease observed in Southern Brazil since 2010^72^.

A drawback to consider about the highlighted agreements and disagreements between the available epidemiological data and our phylodynamic modeling is that while the former characterizes the HIV epidemic of each of the countries/regions as a whole, the C_EA_ clade herein analyzed is not the only prevalent HIV lineage in all of them^5^. Then, it is possible that trends in the number of new HIV cases belonging to the C_EA_ sub-epidemics do not fully correspond with those of the overall HIV epidemic. Besides, a more homogeneous and dense sampling of each C_EA_ sub-epidemic over time as well as the use of sequence data from multiple genetic loci^31^ and the incorporation of covariates into the demographic inference framework^64^ may improve the performance of phylodynamics methods to recover true population trajectories.

Overall, this study supports that major HIV-1 C_EA_ lineages circulating in Eastern Africa and Southern Brazil seem to have had an exponential spread with very similar growth rates until the early/mid-1990s. The overall agreement of the R_0_ and R_e_-initial values here estimated from genetic sequences with those previously obtained from classical epidemiological data strengthen the utility of coalescent and the birth-death phylodynamic approaches to infer relevant epidemiological information of HIV epidemics at the earlier stages. Our data supports that introduction of universal access to ARV therapy during the late 1990s and early 2000s coincides with a declining epidemic in Eastern Africa, but with an upward trend of new HIV diagnoses in Southern Brazil. Our results also underscore the importance of the joint use of both coalescent and birth-death phylodynamic approaches for the analyses of HIV population dynamics given its apparent differential sensitivity for recovering changes in population dynamics at most recent times in different datasets.

## Electronic supplementary material

Supplementary Information

Dataset 1

Dataset 2

Dataset 3

Dataset 4

Dataset 5
