# Supplementary material for: Inferring population dynamics of HIV-1 subtype C epidemics in Eastern Africa and Southern Brazil applying different Bayesian phylodynamics approaches
Source: Sci Rep. 2018 Jun 8;8:8778. doi: 10.1038/s41598-018-26824-4 (PMC5993807; doi:10.1038/s41598-018-26824-4)
Supplement: Supplementary file 1 — Supplementary Information [file 41598_2018_26824_MOESM1_ESM.pdf]

Supplementary Information

Title: Inferring population dynamics of HIV-1 subtype C epidemics in Eastern Africa and Southern Brazil applying different Bayesian phylodynamics approaches.

Authors: Daiana Mir, Tiago Gräf, Sabrina Esteves de Matos Almeida, Aguinaldo Roberto Pinto, Edson Delatorre and Gonzalo Bello

Table S1. HIV-1 subtype C *pol* sequences from Africa and America used for ML phylogenetic analyses.

Region	Country	Total	C_{EA}	Sampling Time
East Africa	Burundi	284	281	2002-2012
	Ethiopia	233	179	2002-2013
	Kenya	55	44	2004-2012
	Rwanda	22	22	2005-2012
	Tanzania	177	76	2003-2014
	Uganda	62	58	1990-2010
South Africa	Zambia	148	0	1998-2008
Central Africa	Democratic Republic of the Congo	22	4	2002-2007
America	Brazil	144	144	1992-2014

Table S2. Best fit demographic model for major country-specific HIV-1 C_{EA} subclades.

Clade	Model	PS Log ML	Models compared	Log BF	SS Log ML	Models compared	Log BF
BI-RW	**Log**	**-37755.2**	-	-	**-37766.1**	-	-
	Expo	-37912.4	Log/Expo	157.2	-37917.5	Log/Expo	151.4
	Expa	-37930.9	Log/Expa	175.7	-37930.9	Log/Expa	164.8
ET1	**Log**	**-9237.7**	-	-	**-9238.0**	-	-
	Expo	-9328.4	Log/Expo	90.7	-9328.8	Log/Expo	90.8
	Expa	-9336.2	Log/Expa	98.5	-9336.5	Log/Expa	98.5
ET2	**Log**	**-8111.9**	-	-	**-8112.2**	-	-
	Expo	-8176.5	Log/Expo	64.6	-8176.7	Log/Expo	64.5
	Expa	-8184.8	Log/Expa	72.9	-8185.2	Log/Expa	73.0
TZ	**Log**	**-7878.2**	-	-	**-7878.5**	-	-
	Expo	-7901.0	Log/Expo	22.8	-7901.3	Log/Expo	22.8
	Expa	-7910.3	Log/Expa	32.1	-7910.9	Log/Expa	32.4
BR HET-S	**Log**	**-19564.8**	-	-	**-19568.3**	-	-
	Expo	-19651.6	Log/Expo	86.8	-19654.2	Log/Expo	85.9
	Expa	-19654.1	Log/Expa	89.3	-19656.8	Log/Expa	88.5

Log marginal likelihood (ML) estimates for the logistic (Log), exponential (Expo) and expansion (Expa) growth demographic models obtained using the path sampling (PS) and stepping-stone sampling (SS) methods. The Log Bayes factor (BF) is the difference of the Log ML between of alternative (H1) and null (H0) models (H1/H0). Log BFs > 1 indicates that model H1 is more strongly supported by the data than model H0.

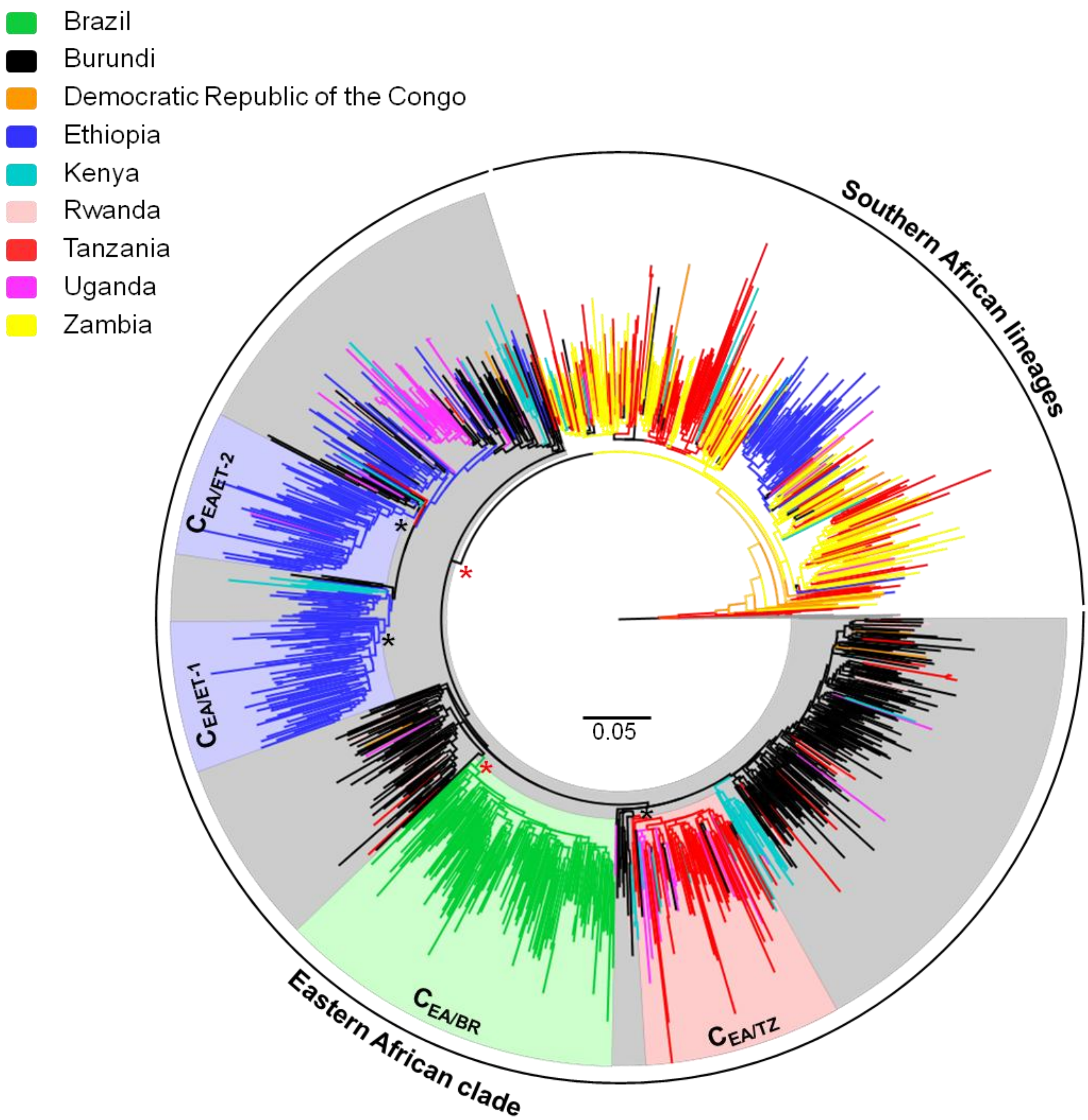

Figure S1. ML phylogenetic tree of HIV-1 C pol PR/RT sequences (~1,000 nt) from east, southern and central Africa and southern Brazil. Branches are colored according to the geographic origin of sequences as indicated in the legend (upper left). Gray shaded box indicate the positions of the Eastern African clade (C_{EA}) and coloured boxes indicate the position of major C_{EA} lineages. Red asterisks point to key nodes with SH-*aLRT* $\geq$ 0.90 and black asterisks point to key nodes with SH-*aLRT* > 0.85. The tree was rooted using HIV-1 subtypes A1 and D reference sequences and the branch lengths are drawn to scale with the bar at the center indicating nucleotide substitutions per site.
